# Multiband transparency effect induced by toroidal excitation in a strongly coupled planar terahertz metamaterial

**DOI:** 10.1038/s41598-021-98498-4

**Published:** 2021-09-28

**Authors:** Angana Bhattacharya, Rakesh Sarkar, Naval K. Sharma, Bhairov K. Bhowmik, Amir Ahmad, Gagan Kumar

**Affiliations:** 1 Department of Physics, Indian Institute of Technology Guwahati, Guwahati, Assam 781039 India; 2 College of Information Technology, United Arab Emirates University, Al Ain, United Arab Emirates

**Keywords:** Optics and photonics, Physics

## Abstract

The multiband transparency effect in terahertz (THz) domain has intrigued the scientific community due to its significance in developing THz multiband devices. In this article, we have proposed a planar metamaterial geometry comprised of a toroidal split ring resonator (TSRR) flanked by two asymmetric C resonators. The proposed geometry results in multi-band transparency windows in the THz region via strong near field coupling of the toroidal excitation with the dipolar C-resonators of the meta molecule. The geometry displays dominant toroidal excitation as demonstrated by a multipolar analysis of scattered radiation. High Q factor resonances of the metamaterial configuration is reported which can find significance in sensing applications. We report the frequency modulation of transparency windows by changing the separation between TSRR and the C resonators. The numerically simulated findings have been interpreted and validated using an equivalent theoretical model based upon three coupled oscillators system. Such modeling of toroidal resonances may be utilized in future studies on toroidal excitation based EIT responses in metamaterials. Our study has the potential to impact the development of terahertz photonic components useful in building next generation devices.

## Introduction

Inducing transparency in an otherwise absorptive spectrum has garnered a considerable interest of the scientific community in the last decade because of its significance in constructing photonic components such as slow light systems, buffers and tunable filters. In this context, plasmon or electromagnetically induced transparency (EIT) has been a prime focus in inducing transparency windows. They are basically a quantum mechanical phenomenon which occurs in three level atomic systems. In these effects, absorption gets cancelled and the medium becomes transparent to a beam of light^1,2^. Within the transparency window, the medium becomes highly dispersive which is important to several applications including, sensing, optical data storage and slow light phenomenon^3,4^. Specific experimental conditions including constraints on temperature and high intensity pumping has led to the exploration of classical alternatives to the EIT phenomenon^5^. The classical analogue of EIT has been rigorously studied experimentally and numerically via metamaterials. Metamaterials (MMs) are artificial structures with exceptional properties not seen in natural media, consisting of subwavelength unit cells known as ‘meta-atoms’. Properties of MMs depend on that of the meta-atoms which can be altered at will via careful design of the resonant meta-atoms^6^. The classical analogue of EIT in MMs is generally discussed via the destructive interference between a dark mode and a bright mode leading to a narrow single band or double band transparency window in an otherwise absorbing region^7–11^. The bright mode has stronger coupling with incident radiation and exhibits broader resonance spectrum and low Q factor. On the other hand, the dark mode has weaker coupling with the incident radiation and has narrower resonance and high Q factor. Reports have also been made on the coupling between bright modes resulting in EIT window. In contrast to bright-dark mode coupling where at EIT peak frequency, the resonator corresponding to the dark mode gets excited, in bright-bright mode coupling both the resonant structures get excited or both get supressed^12–14^.

In recent years, researchers have paid a lot of attention to explore the transparency effects using toroidal excitations in metamaterials. Poloidal currents flowing along the arms of a torus leads to the excitation of end- to-end arrangement of magnetic moments resulting in a toroidal dipolar excitation along the symmetry axis of the torus.They were introduced as a separate family of moments over electric and magnetic moments^15^. While toroidal excitations are supressed by electric and magnetic dipolar excitations in natural materials, MMs can often be designed to predominantly achieve toroidal behaviour displaying signifant low losses and high qualtiy factors^16–20^. The distinctive qualities of toroidal excitations have found applications in switches, sensors, biosensors and other photonic devices^21–23,30^. Investigations on active modulation of toroidal resonances using graphene-based MM devices have also found prominence in recent literature^24,25^. Studies have rigorously explored dielectric toroidal metamaterials, interaction and coupling of toroidal excitations in bilayer metamaterials^26–28^.

Toroidal resonance excitations for their potential in inducing transparency windows and causing steep dispersion at microwave frequencies has been explored in recent times. The toroidal resonances have unique features such as low radiation loss and narrow line width as compared to the electric and magnetic dipolar resonances^29,30^. Therefore, they are very sensitive to the biological perturbations. The high Q factor associated with toroidal excitations makes them interesting for engineering and building the low-loss photonic devices^31–33^. Considering these aspects, the potential of the toroidal excitation induced EIT in the THz spectrum can be immense. It has been observed that the coupling between toroidal and dipolar (electric and magnetic) resonances can result in narrow transparency windows with high Q factor resonances as compared to the dipole-dipole coupled system. This can find applications in the highly sensitive chemical and bio-molecular sensing^34^. Toroidal EIT also exhibit steeper dispersion within the transparency window resulting in an elevated group refractive index which can cause the group velocity of light to reduce significantly in the medium. This property can be useful in making optical devices such as slow light system, switches, optical buffers etc. In this context, Li et al. demonstrated EIT effect in a planar MM in the microwave frequency regime comprising of a toroidal asymmetric SRR acting as a dark mode resonator and cut wires acting as bright modes^35^. Similar work has been reported in literature where single EIT window is observed in the GHz range and multipolar analysis indicates prominence of toroidal excitations for the dark mode resonator^36,37^. Shen et al. observed EIT via combination of an asymmetric elliptical SRR and cut wires^36^. The combination resulted in an EIT window in the GHz range and the combined structure demonstrated toroidal behaviour^38^. Dynamic manipulation of EIT effect has also been reported in a graphene loaded all dielectric meta surfaces^39^. In another study, Shen et al. demonstrated dual band EIT effect in E-$\epsilon$ planar MM structure in microwave frequency regime. In the terahertz (THz) frequency domain, Wang et al. have explored the excitation of single EIT window where mutual coupling of two asymmetric ‘J’ shaped metal rings was observed^40^. Multipolar analysis indicated dominance of toroidal dipolar resonance . Despite the growing interest, no study has been performed to examine multiband transparency effect induced by toroidal dipolar excitations in the THz region to the best of our knowledge. Toroidal excitations strongly coupled with the dipolar resonances of the metamaterials can result in transparency windows that can be manipulated with coupling between the resonators. In our study, we explore multiband transparency effect by coupling between a toroidal resonator and two ‘C’ shaped resonators in the THz frequency regime. A theoretical modeling for toroidal resonance based EIT phenomenon is also explored and reported. We develop a thorough understanding of the coupling mechanism and present a theoretical interpretation.

The paper is organised as follows. We initially discuss the design of our proposed MM unit cell. The results obtained via numerical simulations are elaborated and the multiband EIT effect is explained using the transmission plots and electric field profiles. The surface current profiles indicate toroidal and electric dipolar excitation in each individual resonator. A multipolar analysis confirms the dominance of the toroidal excitations in the MM design. We then provide a theoretical modeling to provide a rigorous understanding of the EIT phenomenon.

**Figure 1:**
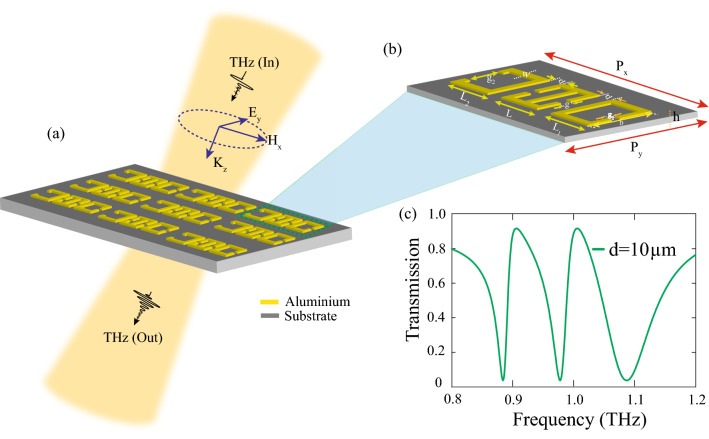
(**a**) Schematic  depicting the terahertz transmission through the proposed MM configurations. The incident THz field is polarized parallel to the split gap i.e, along the y axis. (**b**) Magnified view of the unit cell of the MM comprising of a toroidal SRR and two asymmetric C shaped resonators made up of aluminium. The two C shaped resonators are placed symmetrically on both sides of the TSRR. The dimensions of the unit cell are periodicity ‘ $P_{x}$’ = 130 $\upmu \hbox {m}$, ‘ $P_{y}$’ = 80 $\upmu \hbox {m}$, width ‘ w’ = 4 $\upmu \hbox {m}$. Split gaps ‘ g’ and ‘ $g_{1}$’ = 3 $\upmu \hbox {m}$, ‘ $g_{2}$’ = 21 $\upmu \hbox {m}$. The length of mid SRR (TSRR) is ‘ L’ = 32 $\upmu \hbox {m}$ and that of the other C shaped SRRs (CSRR) is ‘ $L_{1}$’ = ‘ $L_{2}$’ = 30 $\upmu \hbox {m}$. The distance of each CSRR from the mid TSRR is termed as ‘*d*’. (**c**) Transmission spectra showing multiband transparency effect for ‘*d*’= 10 $\upmu \hbox {m}$.

## Design of the MM unit cell

To achieve multiband transparency windows with toroidal resonance, we carefully designed our MM unit cell. The schematic of the proposed MM configuration along with the unit cell are depicted in Fig. 1. Our geometry consists of a split ring resonator with symmetric gaps on each arm flanked by two C shaped resonators on each side. The split ring resonators are made up of aluminium. The MM resonators are designed on a quartz substrate having relative permittivity of $\epsilon _{r}$ = 3.75. The periodicity in the x direction is given by $P_x$ =130 $\upmu \hbox {m}$, that in the y direction is given by $P_y$ = 80 $\upmu \hbox {m}$. The parameters of the unit cell are magnified in the corresponding image of Fig. 1b. The length ‘ L’ of the mid resonator is 32 $\upmu \hbox {m}$, and that of C shaped resonators, ‘ $L_1$’ =‘ $L_{2}$’ = 30 $\upmu \hbox {m}$. The breadth of each resonator ‘ b ’ is taken as 35 $\upmu \hbox {m}$. The capacitive gap ‘ g’ = ‘ $g_{1}$’ is 3 $\upmu \hbox {m}$ for the mid resonator and right ‘ C ’ resonators while the capacitive gap of the left most C shaped resonator is ‘ $g_2$ ’ = 21 $\upmu \hbox {m}$. ‘*d*’ depicts the distance of each C shaped resonator from the mid resonator. The width of the SRRs is given by ‘ w ’ = 4 $\upmu \hbox {m}$. We have summarised the parameters of the MM unit cell in Table 1.

**Table 1:** Dimensional parameters of the MM unit cell.

Parameter	Definition	Value ($\upmu \hbox {m}$) (pH)
Periodicity along x $\times$ direction	$P_{x}$	130
Periodicity along y $\times$ direction	$P_{y}$	80
Length of middle resonator	L	32
Length of ‘C’ resonators on the side	$L_{1} = L_{2}$	30
Breadth of each resonator	b	35
Capacitive gap of mid resoantor	g	3
Capacitive gap of right ‘C’ resoantor	$g_{1}$	3
Capacitive gap of left ‘C’ resoantor	$g_{2}$	21
Width of each resonator	*w*	4

**Figure 2:**
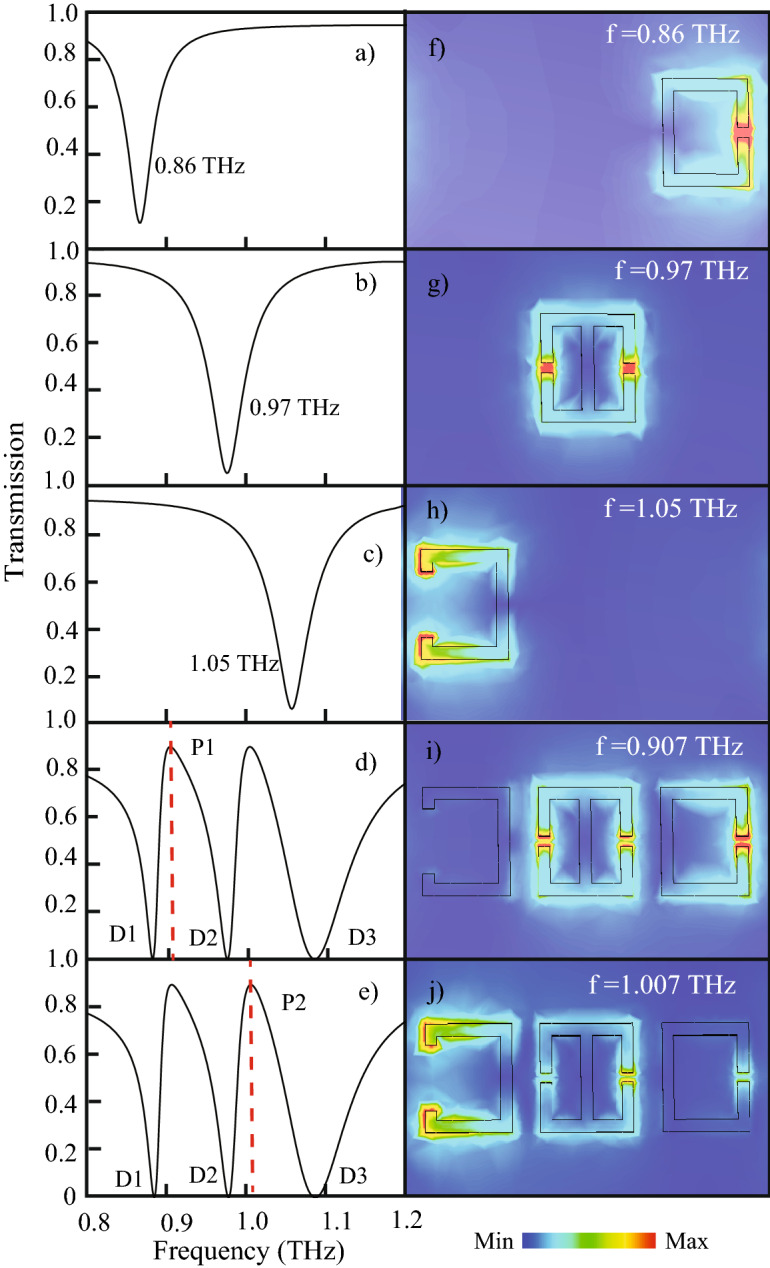
The transmission spectra for (**a**) the right CSRR indictating individual resonance at 0.86 THz (**b**) the mid TSRR demonstrating resonance at 0.97 THz (**c**) the left CSRR showing resonance at 1.05 THz (**d**) and (**e**)  the proposed combined MM configuaration at d = 10 $\upmu \hbox {m}$.  The dotted red lines indicate the transmission profiles at the 1^st^ and 2^nd^ transparency peaks P1 and P2 respectively. Multiband EIT is observed with peak frequencies indicated by red dotted lines at P1 and P2. (**e**) Peak P2 of the EIT window at 1.07 THz. (**f**) Electric field profile showing right CSRR excited at 0.86 THz. (**f**–**h**) show induced electric field profiles for individual (**g**)  TSRR excited at 0.97 THz  left CSRR excited at 1.05 THz. (**i**) electric field profiles at peak P1 (0.907 THz) showing excitation of the TSRR and right SRR indicating coupling between the two. (**j**) Electric field profile corresponding to peak P2 (1.007 THz) showing excitation of left CSRR and TSRR.

Figure 1c shows the transmission plot when both the C shaped resonators are symmetrically placed from the mid resonator by *d* = 10 $\upmu \hbox {m}$. Terahertz radiation is incident on the MM configuration with electric field polarised parallel to the split gaps along the y-direction. CST microwave studio software, version 2020 (http://cst.com), was used for the design and numerical simulations. We used tetrahedral meshing in the frequency domain solver.  Unit cell boundary conditions were set along x and y direction while open boundary condition is used along the direction of the incident light. The geometry can be fabricated via conventional photo-lithography or electron beam lithography in a clean room environment.

## Results and discussions

The transmission for the proposed MM is studied numerically for incident terahertz beam. The transmission spectrum obtained for the configuration with *d*=10 $\upmu \hbox {m}$ is plotted in Fig. 1c. Three resonance dips and two transparency windows are observed. The first resonance dip D1 is at 0.88 THz, the second dip D2 is at 0.979 THz while the third dip is observed at 1.08 THz. The two transparency peaks termed as P1 and P2 are excited at 0.907 THz and 1.007 THz respectively. To understand the multiband EIT behaviour, transmission spectra and electric field profiles of each individual resonators and also that of the combined structure are studied in Figure 2. Figure 2a describes the transmission obtained from the right CSRR. The transmission plot shows a resonance dip at 0.86 THz. The black trace in Fig. 2b depicts the transmission obtained from the mid TSRR. A resonance dip at 0.97 THz is observed. Further, the transmission plot for the left CSRR in Fig. 2c portrays a resonance dip at 1.05 THz. On combining all the three resonators, as observed from Fig. 2d,e, three resonance dips (D1, D2, D3) and two resonance peaks (P1 and P2) are obtained. It may be suggested that the first dip D1 at 0.88 THz is due to the right CSRR, the second dip D2 at 0.979 THz is due to the mid TSRR and the third dip at D3 at 1.08 THz is due to the left CSRR, as can be understood from the behaviour of individual transmission plots of the three resonators. The strong near field coupling between the three resonators result in the transparency window observed in Fig. 2d,e with transparency peaks at P1 corresponding to frequency 0.907 THz and P2 corresponding to frequency 1.007 THz respectively.

To confirm multiband EIT due to coupling between the resonators, we discuss the electric field profiles of the individual resonators and the proposed MM configurations. Figure 2f–h provides a visual understanding of the electric field behaviour for the meta-atom. The electric field profile in Fig. 2f clearly demonstrates that the field is strongly confined at the split gap of the right most resonator at 0.86 THz. From Fig. 2g we observe that the TSRR is excited at 0.97 THz. It is further seen in Fig. 2h that at 1.05 THz, electric field excitation is highest for the left resonator. This behaviour of electric field excitation is in line with the transmission resonance trend we studied for each individual resonator. It can also be stated that each resonator behaves as a bright mode and is directly coupled to the incident terahertz radiation. Next the electric field excitations at the two peaks of the multiband transparency windows are studied. Figure 2i corresponds to the electric field excitation at peak P1 (0.907 THz) while Fig. 2j depicts the field excitation at P2 (1.007 THz). It is observed that at P1, the TSRR and right CSRR are excited strongly. Thus, it can be derived that there is a strong bright-bright mode near field coupling between the two resonators which leads to the introduction of 1st transparency window. Further, A similar observation is made at P2 where the TSRR and left CSRR are strongly excited, which signifies that the strong near field coupling between these two SRRs introduces the 2nd transparency window. The mode hybridization of the TSRR and the C shaped SRRs, and subsequent frequency detuning leads to the two transparency windows. Hence, it can be inferred that strong near field coupling between the bright -bright modes leads to the multiband EIT windows peaking at P1 and P2 respectively^13,14^.

**Figure 3:**
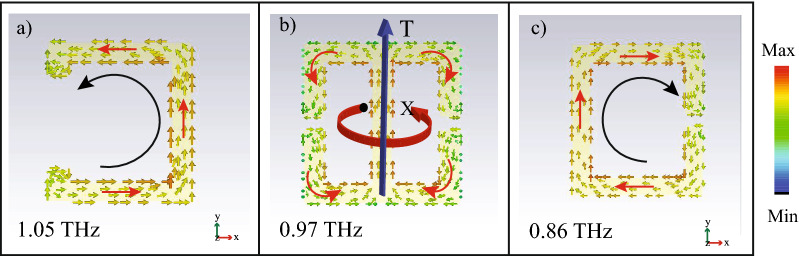
(**a**) Anticlockwise flow of surface current on the left CSRR (1.05 THz) exhibiting electric dipolar excitation. (**b**) Surface current profile in the mid TSRR (0.97 THz) showing end to end formation of magnetic dipole moment leading to toroidal dipolar excitation T. (**c**) Clockwise flow of current in thr right CSRR (0.86 THz) exhibiting electric nature of exciation. Here the green arrow indicates polarization direction of  the incident terahertz light.

Next, we study the nature of the EM excitation in each of the separate SRR by an individual evaluation of the surface current profiles. Figure 3 presents a magnified image of the resonators. Figure 3a exhibits surface current flowing along the anticlockwise direction leading to a dipolar excitation in the left ‘ C’ resonator. Similar behaviour is observed in the right ‘ C’ resonator , demonstrating dipolar excitation with current circulating in the clockwise direction. For the mid resonator shown in Fig 3b, it is observed that clockwise current flows along the right side resulting in magnetic dipole moment going inside the plane of the MM. On the left side of the mid resonator, magnetic moment forms coming out of the plane due to anticlockwise current flow. This end to end formation of magnetic moments , as depicted by the circulating red arrow, result in the excitation of toroidal dipolar moment along the y direction. Thus, the mid SRR exhibits toroidal dipolar excitation and is termed as ‘ TSRR’.

**Figure 4:**
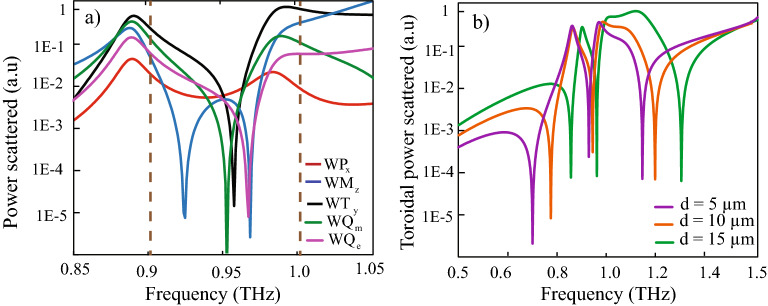
(**a**) Multipolar analysis for the MM configuration over the simulated frequency range indicating a dominance by toroidal dipolar excitation over electric and magnetic dipolar contributions, as well as electric and magnetic quadrupolar contributions. Dotted brown lines indicate positions of peak 1 and peak 2, respectively. (**b**) Blue shift in in the toroidal scattered power on increasing the distance ‘*d*’ between the mid SRR and the C shaped resonators on both sides.

To get a clear idea of the dominant nature of excitation in the combined proposed MM design, a multipolar analysis is performed for the five major electromagnetic moments i.e., electric dipole moment, magnetic dipole moment, toroidal dipole moment and electric and magnetic quadrupole moments^23,41^. The power radiated by each of of the multipoles is evaluated. The power radiated by the individual moment i.e., ‘ WP’,‘ WT’, ‘ WM’, ‘ WQ$_{m}$’, and ‘ WQ$_{e}$’ respectively, for electric, toroidal, magnetic dipolar moment and magnetic and electric quadrupole moment is given by,

1 $$WP = \dfrac{2\omega ^{4}}{3c^{3}}{} \mathbf |P| ^{2},$$

2 $$WT = \dfrac{2\omega ^{6}}{3c^{5}}{} \mathbf |T| ^{2},$$

3 $$WM = \dfrac{2\omega ^{4}}{3c^{3}}{} \mathbf |M| ^{2},$$

4 $$WQ_{m} = \dfrac{\omega ^{6}}{40c^{5}}{\Sigma |\mathbf{MQ}_{\alpha \beta }|}^{2},$$

5 $$WQ_{e} = \dfrac{\omega ^{6}}{5c^{5}}{|\mathbf{EQ}_\alpha \beta |}^{2},$$

where the angular frequency of incident radiation is represented by $\omega$, speed of light in vacuum is given by c. P, T , M, $MQ_{\alpha \beta }$ and $EQ_{\alpha \beta }$ represents the electric, toroidal, magnetic dipolar moments, and the magnetic and electric quadrupole moments respectively, calculated using the surface current data. The black line in Fig. 4a represents the power radiated by toroidal dipolar moment, the blue line depicts the contribution to radiated power by magnetic dipolar moment. The scattered power contribution by the electric dipolar moment is shown by the red line. The green line shows power scattered by magnetic quadrupole moment while the purple line shows power scattered by electric quadrupole moment. The brown dotted lines indicate the position of peak 1 and peak 2 of the multiband windows. We observe significant contribution from toroidal scattered power and magnetic quadrupole scattered power near the peak frequencies of the EIT windows.The black curve at the peak frequencies of transparency windows, i.e., P1 and P2, and also at 0.97 THz, which is the resonance frequency of the TSRR, shows higher value of toroidal scattered power when compared to the other curves. It is evident from the figure that there is a dominant contribution to scattered power by the toroidal dipolar excitation in both the peak frequencies, P1 and P2. This confirms the toroidal nature of the mid resonator and also, the prominent dominance of toroidal excitation in the MM design in the EIT window. It signifies the excitation of electromagnetically induced transparency effect due to a toroidal terahertz MM configuration. We further examine the behaviour of the scattered power by toroidal dipolar moment on changing the distance ‘ *d*’ between the TSRR and CSRR symmetrically, as depicted in Fig. 4b. The violet line indicates power scattered for ‘ *d*’ = 5 $\upmu \hbox {m}$, the orange line for ‘ *d*’ = 10 $\upmu \hbox {m}$ and the green line signifies power scattered for ‘*d*’ = 15 $\upmu \hbox {m}$ respectively. A blue shift in the scattered power is observed on the increase of *d*.

To get a better idea of sensing capacity of our proposed MM, the Q factor of the ‘ *d*’ = 10 $\upmu \hbox {m}$ configuration for the first and second dips are calculated using the formula $Q=\frac{f_{0}}{FWHM}$, where $f_{0}$ is the resonant frequency and FWHM is the full width at half maximum of the resonance. It is found that for the first dip Q = 42, while for the second dip, Q = 34. Such high Q factor toroidal resonance could find utilisation in toroidal EIT based sensor applications.

### Frequency modulation of transparency windows

**Figure 5:**
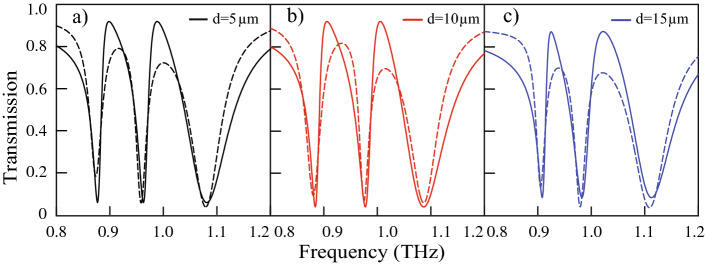
(**a**) Numerically simulated and theoretically fitted transmission spectra for d= 5 $\upmu \hbox {m}$ (**a**), 10 $\upmu \hbox {m}$ (**b**) and 15 $\upmu \hbox {m}$ (**c**). The dotted lines indicate the transmission profile obtained by theoretical modeling for the proposed multiband EIT effect.

The behaviour of the proposed meta-surface on changing the distance between the TSRR and the other two CSRRs is examined. Distance ‘ *d*’ is changed symmetrically with respect to the TSRR by 5 $\upmu \hbox {m}$, 10 $\upmu \hbox {m}$ and 15 $\upmu \hbox {m}$ respectively. Figure 5a shows the transmission plot for *d* = 5 $\upmu \hbox {m}$, while Fig. 5b,c shows the transmission for *d* = 10 $\upmu \hbox {m}$ and *d* = 15 $\upmu \hbox {m}$ respectively. Here the solid curve illustrates the numerically simulated transmission spectra and the theoretically fitted transmission spectra are depicted by the dashed curves. The theoretical model is discussed in the next section. A blue shift in the peak frequencies P1 and P2 can be observed on increasing *‘d'*. Thus, a frequency modulation of the multiband EIT window is achieved by a symmetric increase of the distance between the TSRRs and the CSRRs on each side. It is believed that the near field coupling between the TSRR and CSRRs decreases due to an increase in their separation resulting in a blue shift of the transparency windows in the proposed multiband transparency effect.

## Theoretical modeling

To study the coupling mechanism between the toroidal resonator and two CSRRs and to validate the numerically obtained transmission spectra, we have used a theoretical model based on three coupled harmonic oscillator systems. Such a system can be represented by the following set of equations^42,43^,

6 $$\begin{aligned}{} &\ddot{x}_{1}+\gamma _{1}\dot{x_{1}}+{\omega _{1}}^{2}x_{1}+{\Omega _{1}}^{2}x_{2}+ {\Omega _{2}}^{2}x_{3} = \frac{Q_{1}}{M_{1}} E,\\&\ddot{x}_{2}+\gamma _{2}\dot{x_{2}}+{\omega _{2}}^{2}x_{2}+{\Omega _{1}}^{2}x_{1} = \frac{Q_{2}}{M_{2}} E,\\&\ddot{x}_{3}+\gamma _{3}\dot{x_{3}}+{\omega _{3}}^{2}x_{3}+{\Omega _{2}}^{2}x_{1} = \frac{Q_{3}}{M_{3}} E, \end{aligned}$$

Here we represent the toroidal SRR as oscillator 1, the left CSRR as oscillator 2 and right CSRR as oscillator 3. Also ($x_{1}$, $x_{2}$, $x_{3}$), ($\gamma _{1}$,$\gamma _{2}$ ,$\gamma _{3}$ ), ($\omega _{1}$ ,$\omega _{2}$ ,$\omega _{3}$) are the displacements, loss factors and resonance frequencies of oscillators 1, 2 and 3 respectively. $\Omega _{1}$ and $\Omega _{2}$ represent the coupling strengths between oscillators 1 and 2 and oscillators 1 and 3, respectively. However, we have neglected the coupling between oscillators 2 and 3. ($Q_{1}$, $Q_{2}$, $Q_{3}$), ($M_{1}$,$M_{2}$, $M_{3}$) are the effective charges and masses of the oscillators. The incident electric field of the terahertz radiation is represented by $E=E_{0} e^{i\omega t}$ , where $\omega$ depicts frequency of the incident radiation.

To solve Eq. (6) and get displacements ($x_{1}$, $x_{2}$, $x_{3}$) we have assumed a trial solution as $x_{n}=N_{n}e^{i\omega t}$. By solving Eq. (6) for $x_{1}$, $x_{2}$, $x_{3}$ the susceptibility $\chi$ can be related with the polarization P of incident terahertz radiation as

7 $$\begin{aligned} \chi & = \dfrac{P}{\epsilon _{0}E} = \dfrac{Q_{1}x_{1}+Q_{2}x_{2}+Q_{3}x_{3}}{\epsilon _{0}E}\\& =\,\dfrac{Q_{1}^{2}}{M_{1}\epsilon _{0}}(\dfrac{\alpha _{1}\Omega _{1}^{4}+\alpha _{2}\Omega _{2}^{4}+\alpha _{3}\Omega _{1}^{2}+\alpha _{4}\Omega _{2}^{2}+\eta +\beta \Omega _{1}^{2}\Omega _{2}^{2})}{D_{1}D_{2}D_{3}-\Omega _{2}^{4}D_{2}-\Omega _{1}^{4}D_{1}} \end{aligned}$$

where $D_{1}=\omega _{1}^{2}-\omega ^{2}+i\omega \gamma _{1}$, $D_{2}=\omega _{2}^{2}-\omega ^{2}+i\omega \gamma _{2}$, $D_{3}=\omega _{3}^{2}-\omega ^{2}+i\omega \gamma _{3}$ and $A_{1}=\frac{Q_{1}}{Q_{2}}$, $A_{2}=\frac{Q_{1}}{Q_{3}}$ , $B_{1}=\frac{M_{1}}{M_{2}}$ and $B_{2}=\frac{M_{1}}{M_{3}}$.

Also we have taken here $\alpha _{1}=-\frac{B_{2}}{A_{2}^{2}}$, $\alpha _{2}=-\frac{B_{1}}{A_{1}^{2}}$, $\alpha _{3}=-\dfrac{D_{3}}{A_{1}}(1+B_{1})$, $\alpha _{4}=-\dfrac{D_{2}}{A_{2}}(1+B_{2})$, $\beta =\dfrac{B_{1}+B_{2}}{A_{1}A_{2}}$, $\eta =D_{2}D_{3}+\dfrac{B_{1}D_{2}D_{3}}{A_{1}^{2}}+\dfrac{B_{2}D_{1}D_{2}}{A_{2}^{2}}$.

Theoretically fitted transmission spectra has been obtained by using Krammer–Koning relations $T=1-imag(\chi )$ , where we have used energy conservation principal A + T = 1 (normalized to unity). Here absorption in the medium has been defined by $A=imag(\chi )$ .The dash curves in Fig. 5 illustrate theoretically fitted transmission spectra for *d*=5 $\upmu \hbox {m}$, 10 $\upmu \hbox {m}$ and 15 $\upmu \hbox {m}$. It is evident from the figure that the analytically fitted transmission spectra are in good agreement with the corresponding numerically simulated ones. Here black traces illustrate multiband transparency spectra for *d*=5 $\upmu \hbox {m}$, while that for *d*=10 $\upmu \hbox {m}$ and *d*=15 $\upmu \hbox {m}$ are depicted by red and blue traces respectively. It can be visualized from the figure that as the separation ‘ *d*’ increases, both peaks of the two transparency windows get blue shifted. In the model we have taken resonance frequencies of the three resonators as ($\omega _{1},\omega _{2},\omega _{2}$=0.95 THz, 0.87 THz and 1.08 THz) and kept them constant to theoretically fit the transmission spectra with the simulated ones. The loss factors for three resonators are taken as 0.027 THz, 0.023 THz  and 0.05 THz respectively. Theoretically fitted transmission spectra for *d*=5 $\upmu \hbox {m}$, matches well with the simulated ones for the coupling parameters $\Omega _{1}$=0.25 THz and $\Omega _{2}$ =0.20 THz. A change in the coupling parameters ($\Omega _{1}$, $\Omega _{2}$) leads to the blue shift of the transparency windows with change in ‘ *d*’. The coupling parameters are taken as ($\Omega _{1}$ =0.22 THz, $\Omega _{2}$ =0.18 THz) for *d*=10 $\upmu \hbox {m}$ and ($\Omega _{1}$ =0.20 THz, $\Omega _{2}$ =0.16 THz) for *d*=15 $\upmu \hbox {m}$ respectively. Hence, the blue shift of the transparency peaks with the change in separation ‘ *d*’ is dictated by the reduction of coupling parameters $\Omega _{1}$ and $\Omega _{2}$. A slight variation in the FWHM is observed as we increase d from 5 μm to 10 $\upmu \hbox {m}$ and then from 10 μm to 15 $\upmu \hbox {m}$ for both the peaks with opposite trends. Further investigations through numerical simulations indicate that this slight variation cause only a maximum change of about 3 units in the Q factor.

## Conclusions

We examined multiband transparency effect via strong near field coupling between a toroidal resonator and two CSRRs. This effect in terahertz domain in a toroidal MM configuration has not been reported in literature to the best of our knowledge. The electric field profiles indicate coupling of bright-bright modes leading to multiband EIT with peaks at 0.907 THz and 1.007 THz. A multipolar analysis has been performed demonstrating the dominance of toroidal dipolar excitation in the MM. Frequency modulation of the transparency windows by varying the distance ‘ *d*’ between the adjacent resonators demonstrates a blue shift in the multiband transparency windows with increasing distance, which can be attributed to decreased coupling between the SRRs. For a thorough analytical understanding, the numerical results are fitted with a theoretical model based on three coupled oscillators. It has been found that the 1st coupling parameter $\Omega _{1}$ reduces from 0.25 THz to 0.20 THz and the 2nd coupling term $\Omega _{2}$ reduces from 0.20 THz to 0.16 THz as the separation ‘ *d *’ increases from 5 μm to 15 $\upmu \hbox {m}$. This reduction in coupling parameters signifies that the coupling between the resonators decreases with the increase in ‘ *d*’ leading to the blue shift of the transparency windows. High Q factor (42) of the toroidal resonance indicates potential usage of the MM design in EIT based toroidal sensors. The theorectical modeling could impact future analysis of toroidal MM designs and studies . Such toroidal excitation based transparency effect in MMs would help the design of terahertz multiband devices including sensors, filters and modulators with lower radiation losses.
